# A Comparative Analysis between Active and Passive Techniques for Underwater 3D Reconstruction of Close-Range Objects

**DOI:** 10.3390/s130811007

**Published:** 2013-08-20

**Authors:** Gianfranco Bianco, Alessandro Gallo, Fabio Bruno, Maurizio Muzzupappa

**Affiliations:** Department of Mechanical Engineering, University of Calabria, Via P. Bucci 46/C–Rende, Cosenza 87036, Italy; E-Mails: alessandro.gallo@unical.it (A.G.); f.bruno@unical.it (F.B.); muzzupappa@unical.it (M.M.)

**Keywords:** 3D reconstruction, underwater imaging, active and passive 3D techniques

## Abstract

In some application fields, such as underwater archaeology or marine biology, there is the need to collect three-dimensional, close-range data from objects that cannot be removed from their site. In particular, 3D imaging techniques are widely employed for close-range acquisitions in underwater environment. In this work we have compared in water two 3D imaging techniques based on active and passive approaches, respectively, and whole-field acquisition. The comparison is performed under poor visibility conditions, produced in the laboratory by suspending different quantities of clay in a water tank. For a fair comparison, a stereo configuration has been adopted for both the techniques, using the same setup, working distance, calibration, and objects. At the moment, the proposed setup is not suitable for real world applications, but it allowed us to conduct a preliminary analysis on the performances of the two techniques and to understand their capability to acquire 3D points in presence of turbidity. The performances have been evaluated in terms of accuracy and density of the acquired 3D points. Our results can be used as a reference for further comparisons in the analysis of other 3D techniques and algorithms.

## 1. Introduction

The use of 3D imaging techniques in underwater applications is increasing, in fields such as the survey of submerged artefacts [1], the monitoring of marine flora and fauna [2,3] or the mapping of large areas [4]. Multi-view 3D reconstruction is gaining popularity in underwater photogrammetry [5], as it requires just an off-the-shelf camera (movie or still) to acquire a sequence of overlapped pictures of the scene under ambient or artificial lighting (passive technique). The 3D reconstruction is performed through the identification of common natural features in the image set: therefore, the accuracy of the reconstruction depends on the quality of images and textures. The main problem in underwater imaging is the poor visibility due to scattering effects [6,7], which influences the image quality and limits the application of imaging techniques in close-range measurements. In particular, the active techniques rely on a local acquisition (*i.e.*, based on a sheet or a spot) to minimize the effects of scattering. Therefore, new solutions should be developed in order to improve the 3D acquisition process. Recently, an active stereo technique based on the projection of structured light has been tested experimentally in the laboratory in turbid water [8]. The use of a whole-field structured-light technique allowed for the reconstruction of 3D scenes regardless of the texture, obtaining acceptable results in the close-range digitization of small objects. The main goal of this work is to compare the performance of this active technique with a passive multi-view technique tested in an underwater environment [9]. We want to understand if the use of structured light under water can improve the acquisition with respect to the passive approach in terms of accuracy and density of the acquired 3D points, in order to provide useful information for the design of a new underwater 3D imaging system and to gather a reference dataset to be compared with the results obtained with other algorithms and techniques. The comparison is performed under poor visibility conditions produced in the laboratory by suspending different quantities of clay in a water tank, regardless of the corrections in the optical model of image formation, which is required to deal with refractive or scattering effects. Moreover, we do not take into account other problems that occur in real world applications, like the presence of moving elements (mainly flora and fauna present in the marine environment), the colour alteration, or the instability of the imaging system that should require the synchronization of camera shutters. For a fair comparison, a stereo configuration has been adopted for both the techniques, and we have used the same setup, working distance, calibration and objects.

In the next section, titled Related Works, we provide a brief review of 3D stereo techniques usually employed in underwater environment, then in Active and Passive Stereo Techniques section we describe in detail the 3D stereo reconstruction based on both active and passive approaches. Subsequently, in the Experimentation section, the setup and the experimental tests conducted in laboratory will be described; in the section titled Results Analysis the results are discussed, while comments about the comparison between the two techniques are reported in the Discussion section. Conclusions and Remarks are reported in the last section.

## 2. Related Works

In this section we provide a concise review of underwater 3D optical techniques, classifying them according to their approach (passive and active) and paying particular attention to stereo systems, based on the correspondence problem that can be solved by adopting both active and passive approaches. For a more complete review of underwater imaging techniques see also [10,11]. Underwater calibration is treated at the end of this section.

### 2.1. Passive Techniques

Optical imaging techniques for 3D data acquisition used in underwater environments are based on multiple acquisitions of the scene taken from different viewpoints with a movie or still camera. In these techniques, artificial lights (lamps and spotlights, for example) are used just to illuminate the scene if needed, and are not employed in the triangulation of the 3D points, which, in turn, is based on the knowledge of similar points in the image sequence, found through stereo matching algorithms.

Stereo systems use two digital calibrated cameras to capture the scene; they may be installed on underwater robots in applications like seabed mapping [12], robot localization, and tracking [13]. The use of three synchronized cameras [14] represents a good compromise between accuracy and device dimensions, where the underwater survey has to be conducted by a scuba diver. Movie or still cameras, lodged in water-proof housings, are used in seabed mapping [15] and surveys of archaeological sites [16]. Structure-from-motion techniques are used to reconstruct a scene from a sequence of overlapping images acquired by a single moving camera, for example in coral reef survey [2]. The process is based on the automatic extraction of points of interest (a sparse set of features, such as corners), the tracking of this sparse set of features across the image sequence, and the estimation of their 3D positions using multiple views.

### 2.2. Active Techniques

Underwater active imaging is seriously compromised by scattering and absorption in the medium, that reduces image contrast and light intensity, discouraging the use of a whole field approach in submarine applications. To reduce these limitations, active imaging systems for underwater 3D acquisition can rely on four hardware solutions: polarization, spatial, time and spectrum discrimination [17]. It is possible to improve the image contrast by using a pair of images taken through a polarizer from different orientations [18] or by reducing the backscattering volume by increasing the separation distance between the light source and the sensor [19]. Sophisticated laser-based methods employ narrow beams to reduce the degrading effect caused by the scattering component that appears in the overlapped volume of the device FOVs [20], while Laser Line Scan methods, based on triangulation, involve the optical scanning of a narrow FOV [21]. The time discrimination approach rejects the noise component with a gated receiver that confines the instantaneous scattering of a small volume of water close to the target [22]. Finally, the light absorption can be reduced by the selection of a green/blue wavelength for the light source.

Underwater 3D techniques can be based on triangulation and time-of-flight. Both of these principles adopt a local approach to scan the entire target of interest, using a sheet light or a narrow beam. The single sheet projection is useful in several applications like underwater navigation [21], pipe inspection [23] and mapping of underwater archaeological sites [24]. Finally, we have to mention the confocal imaging technique, used to improve underwater imaging in the presence of turbidity [25].

### 2.3. Stereo Matching

Stereo matching algorithms are generally classified based on whether they use local or global methods [26], and both categories are based on some constraints used to solve the correspondence problem: similarity constraint, epipolar constraint, uniqueness constraint, continuity constraint, and ordering constraint. Local algorithms (window based and feature based) enforce these constraints on a limited number of pixels that surround the pixel to be matched. Global algorithms enforce constraints on the whole epipolar line, passing through the pixel or on the entire image. Local algorithms are typically faster than global approaches and more suited for hardware/real-time implementation, but in most cases they are outperformed in terms of accuracy by global approaches.

A different approach involves the projection of a sequence of structured-light patterns in order to codify the scene to be acquired. In this way, the matching of the points is performed automatically, because each point object is uniquely determined by an assigned code [27]. This technique will be described in detail in the next section.

### 2.4. Underwater Calibration

A calibration procedure is required for the extraction of 3D metric measurements from a set of digital images. Several algorithms and methods for camera calibration, generally based on perspective or projective camera models, have been developed and discussed in the literature. The calibration of an underwater optical device must take into account the effect of refraction at the air-acrylic and acrylic-water interfaces (planer or hemispherical, so-called planar and dome port, respectively), which are present when a camera is mounted in its housing [28–30]. The refraction can be considered through the explicit modelling of the optical paths using ray tracing [13] or with the implicit incorporation of the refraction effect into camera calibration parameters [14]. In the first case, the cameras are calibrated in air, and then calibrated in water to derive the geometry of the refractive interfaces, since the principal component of both refractive effect and image distortion is radial [15]. Although the assumption on the refractive indices for the acrylic dome port and the internal air path is reliable, the changes in temperature, pressure, and salinity of the water can cause small variations in the refractive index of the media that cannot be avoided. The alternative implicit approach incorporates refractive effects of the optical components and refractive interfaces into camera calibration parameters.

## 3. Active and Passive Stereo Techniques

In this section, we describe the methods and algorithms employed in the two 3D imaging techniques used in the experimentation. In particular, a stereo configuration has been adopted for a fair comparison between the active technique experimented in [8] that uses structured-light and stereo acquisition, and a passive one with the same setup, working distance, calibration, objects, and lighting conditions. In this way, the acquired 3D point clouds are computed with respect to the same coordinate system; this allows us to align them automatically, limiting the errors due to the registration process.

### 3.1. Optical Model

**Figure 1.**
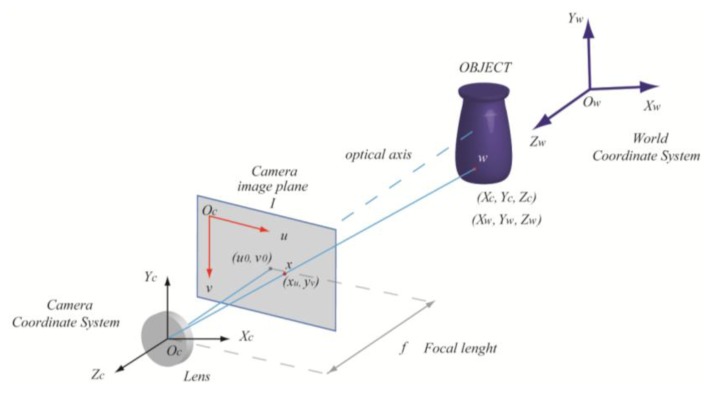
Pinhole camera model. The image point **x** = (x_u_, y_v_) of a 3D point w is the intersection of the optical ray going through the optical centre O_c_ and the image plane I at a distance equal to the focal length f.

The pinhole camera model is used to describe the geometry of the optical devices. Considering the perspective projection of an object point w (Figure 1) with coordinates **X_c_** = [X_c_, Y_c_, Z_c_]^T^ in the camera coordinate system onto the image plane I with coordinates **x** = [x_u_, y_u_]^T^ we have:

(1) $$\text{x}=\frac{\text{f}}{{\text{Z}}_{\text{c}}}\left[\begin{array}{c} {\text{X}}_{\text{c}}\\ {\text{Y}}_{\text{c}} \end{array}\right]$$

where f is the focal length. We can relate **X***_c_* with coordinates **X***_w_* = [*X_w_*, *Y_w_*, *Z_w_*]^T^ in the world coordinate system through a rigid-body transformation:

(2) $${\text{X}}_{\text{c}}=R{\text{X}}_{w}+\text{T}$$

where *R* is the rotation matrix and **T** the translation vector. By introducing the homogeneous coordinates for the vectors **x**, **X_c_**, and **X_w_** from Equations (1) and (2) in matrix notation, we get:

(3) $$\text{x}=P{\text{X}}_{\text{w}}$$

where P is the Perspective Projection Matrix (PPM) representing the geometric model of the camera. In a real camera, the lens produces optical distortions with radial and tangential components, which must be taken into account in the camera model. This way the real (distorted) image coordinates **x_d_** = [x*_d_*, y*_d_*] are related to the ideal (undistorted) image coordinates **x** = [x_u_, y_u_] through the relations:

(4) $$\begin{array}{cc} x_{d}=x_{u}+k_{x}(x_{u},y_{u}) & y_{d}=y_{u}+k_{y}(x_{u},y_{u}) \end{array}$$

where k_x_(x_u_, y_u_) and k_y_(x_u_, y_u_) are the distortion coefficients. Finally, it must be considered that a digital camera involves measures in terms of pixels, through an affine transformation which takes into account the translation of the principal point and a scaling along u and v axis in the image plane:

(5) $$\begin{array}{cc} u=s\frac{x_{d}}{d_{x}}+u_{0} & v=s\frac{y_{d}}{d_{y}}+v_{0} \end{array}$$

where s is a scale factor, (u_0_, v_0_) is the principal point location in pixel coordinates and (d_x_, d_y_) are the pixel dimension in the u and v direction, respectively.

### 3.2. 3D Stereo Reconstruction

**Figure 2.**
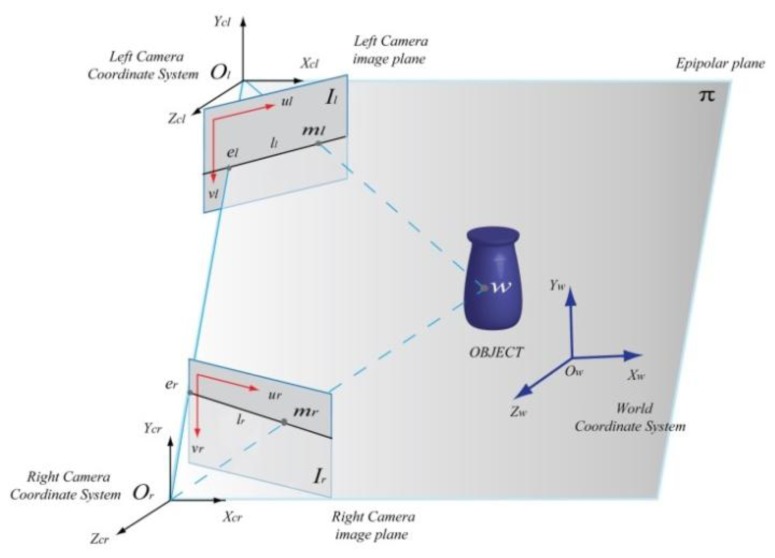
Epipolar geometry. The points m_l_ and m_r_, located in left and right images, respectively, are the projection on the image planes I_l_ and I_r_ of the same 3D point w of the object. The intersections of the line (O_l_, O_r_) (baseline) with each image plane are called epipoles (e_l_ and e_r_). The lines l_l_ and l_r_ are called epipolar lines, intersection of the epipolar plane p (O_l_, O_r_, w) with the two image planes.

Given two images acquired from slightly different viewpoints, a stereo matching algorithm tries to identify the corresponding points (solving the so-called correspondence problem) in both the images related to the same scene point (Figure 2). Knowing these correspondences and the camera geometry, the 3D world coordinates can be reconstructed by triangulation [31].

Given a point m_l_ in the left image, the correspondent point m_r_ in the right image is constrained to lie on the epipolar line (epipolar constraint). Using the perspective projection theory (see Equation (3)), the coordinates of the correspondent points ml and m_r_ can be defined through the relations [31]:

(7) $$\{\begin{array}{c} {\text{m}}_{\text{l}}=P_{l}{\text{X}}_{\text{w}}\\ {\text{m}}_{\text{r}}=P_{r}{\text{X}}_{\text{w}} \end{array}$$

where P_l_ and P_r_ are the PPMs of the left and right camera, respectively. Considering that the point m_l_ (conjugate of m_r_) lies on the epipolar line LL, we can write the following relation (the Longuet-Higgins equation):

(8) $$m_{l}^{T}Fm_{r}=0$$

where F is the Fundamental Matrix, obtained from the stereo calibration process, and depends on the epipolar geometry and the two PPMs.

The last equation solves linearly F using at least eight conjugated points up to a scale factor: with more than eight points, a least-squares solution is found, so the pair of PPMs can be computed from F. Given these two camera matrices and the pairs of points m_l_ and m_r_ that satisfy the epipolar constraint, the 3D coordinates of w are computed by triangulation. Then, finding the intersection of the optical rays corresponding to the two conjugated points m_l_ and m_r_ is a problem that can be formulated as an over-determined equation system. If we express the matrix P_l_ according to its rows and include it in the perspective projection Equation (3), we obtain:

(9) $$m_{l}=\left|\begin{array}{c} {p_{l}}_{1}^{T}\\ {p_{l}}_{2}^{T}\\ {p_{l}}_{3}^{T} \end{array}\right|w$$

and then we write the coordinates of the point m_l_ in the left image plane I_l_ as:

(10) $$\{\begin{array}{c} u_{l}=\frac{{p_{l}}_{1}^{T}w}{{p_{l}}_{3}^{T}w}\\ v_{l}=\frac{{p_{l}}_{2}^{T}w}{{p_{l}}_{3}^{T}w} \end{array}$$

that represent the perspective projection Equation (3) in Cartesian coordinates. By writing the Equation (10) also for the conjugate right image point m_r_, we obtain a homogeneous linear system of the type A·w = 0 that can be solved by the least-squares method to compute the 3D world coordinates of the point w given the coordinates of a pair of correspondent points m_l_ and m_r_.

To simplify the search of corresponding points, the images are commonly rectified (epipolar rectification), putting the stereo rig in a more convenient configuration in which both epipoles are located at infinity and the epipolar lines form a sheaf of parallel lines in both left and right images. In this case each pair of correspondent points are constrained to lie on the same image row (scan-line) so that the correspondence problem is reduced to a one-dimensional search along each epipolar line.

The disparity, that encodes the depth of the scene, represents the distance between x-coordinates or a pair of correspondent points in left and right (rectified) images. Finding a pair of correspondent points is not so trivial. Since the scene is acquired from different points of view, it is possible to find false correspondences due to occlusion, radiometric distortion and perspective distortion.

### 3.3. System Calibration

A calibration procedure is needed to compute the intrinsic parameters of each camera (focal length, coordinates of the principal points, radial and tangential distortions, pixel size) and the extrinsic parameters (translation and rotation with respect to a world coordinate system) of camera and stereo system. These parameters are computed using the well-known Camera Calibration Toolbox for Matlab [32]. The intrinsic and extrinsic parameters of each camera are obtained by correlating the coordinates of known points located on a calibration sample (*i.e.*, a checkerboard) with the corresponding coordinates on the image plane. The next step is to compute the extrinsic parameters of the system (stereo calibration), relating each camera frame to a unique world coordinate system. In this way a relationship between the world coordinate system and the left and right camera coordinate systems is established.

### 3.4. Passive Approach

A Patch-based Multi-View Stereo (PMVS) algorithm, widely used in air applications, has been employed to solve the correspondence problem on a calibrated image pair [33]. Recently, PMVS has also been used for 3D reconstructions of archaeological artifacts in deep water [9]. This open source software, typically employed for multiple-views 3D reconstruction, has been used in a stereo configuration in the laboratory tests, in order to conduct a fair comparison with the active technique. The algorithm estimates the surface orientation while enforcing/strengthening the local photometric consistency, which is important to obtain accurate models from low textured objects or from images affected by blur due to turbidity in underwater environment. PMVS outputs a dense collection of small oriented rectangular patches (a local tangent plane approximation of a surface), and consists of a three-step procedure (matching, expanding, filtering). In the first one, a sparse matching algorithm based on Harris and DoG (Difference-of-Gaussian) operators, detects and matches a collection of reliable point features, sparsely distributed in both images, that satisfy the epipolar constraint. In the expansion step, these initial matches are propagated to the neighboring pixels, in order to obtain a dense collection of patches. Finally, in the last step, false matches are deleted using the visibility constraint. The number of 3D points calculated is strictly related to image resolution, image quality (contrast, focus, *etc.*) and object surface properties, *i.e.*, a low textured object can generate false correspondences and a lesser number of points.

### 3.5. Active Approach

**Figure 3.**
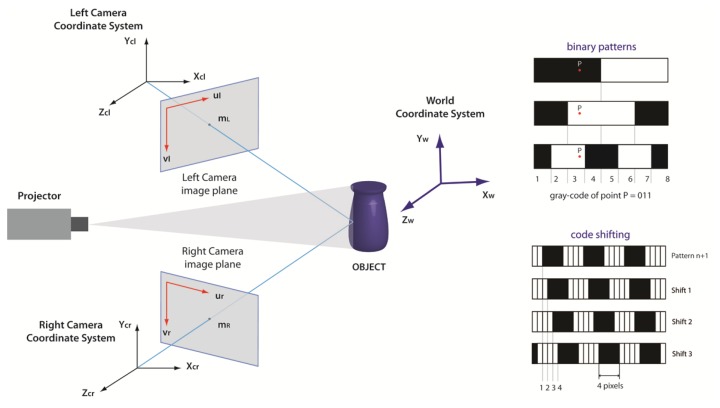
(**Left**): triangulation of a stereo configuration composed by two cameras and a projector. The points m_L_ and m_R_ are the projection of the same 3D point object w on the image planes of the two cameras. (**Right**): examples of binary patterns and code shifting.

The active stereo technique used in this work is based on the codification of a black/white pattern set projected on the object through a digital projector. In particular, the grey-code technique generates a more effective matching between correspondent points in stereo pairs [27]. The object is illuminated by a set of *n* temporally encoded patterns of black/white bands, with a progressively halved width, so that *n* images are captured by each camera. A binary code (*n* bit) is assigned to each point of the image, and the values 0 and 1 are associated to intensity levels, *i.e.*, 0 = black and 1 = white (see Figure 3). This procedure allows to codify 2*n* − 1 lines, defined as crossing zones between white and black bands. Moreover, coded patterns with a bandwidth of four pixels, shifted in steps of one pixel for a total of four pattern positions, are used to exploit the minimum resolution of the projector. By projecting both horizontally and vertically striped coded patterns, a double code is assigned to the intersection points through horizontal and vertical lines. This procedure allows us to codify automatically each point of the object surface. In this work a set of eight vertical and eight horizontal patterns (8-bit code) is used for grey-code. Other 4 + 4 patterns are projected for vertical and horizontal code shifting, respectively, with a bandwidth of four pixels. A projector resolution of 800 × 600 points allows to codify 799 lines × 599 lines = 478,601 points. The projector is only used to establish the correspondences and is not involved in the triangulation, so the calibration of its optics is not necessary. In contrast to traditional passive approaches, this technique does not rely on images with consistent textures, because each point on the object surface is precisely identified by a double binary code.

### 3.6. Calibration

A calibration procedure is needed to compute the intrinsic parameters of each camera (focal length, coordinates of the principal points, radial and tangential distortions, pixel size) and the extrinsic parameters (translation and rotation with respect to a world coordinate system) of camera and stereo system.

The camera calibration procedure is based on an implicit approach. The intrinsic and extrinsic parameters of each camera are obtained by correlating the coordinates of n known points w_i_ located on a calibration sample with the corresponding coordinates m_i_ = [u_i_, v_i_] on the image plane. By writing the Equation (10) in matrix form for this set of n known points:

(11) $$\left[\begin{array}{c} \begin{array}{ccc} w_{i}^{T} & 0 & -u_{i}w_{i}^{T} \end{array}\\ \begin{array}{ccc} 0 & -w_{i}^{T} & v_{i}w_{i}^{T} \end{array} \end{array}\right]\;\left[\begin{array}{c} p_{1}\\ p_{2}\\ p_{3} \end{array}\right]=0_{2x1}$$

we obtain a set of 2n homogeneous linear equations that can be solved via the least-squares method. In theory, six non-coplanar points are sufficient to compute P, but in practice more points are necessary to compensate measure errors due to image noise, for example. This method is the well-known Direct Linear Transformation (DLT) [34], a quite fast process that minimizes an algebraic error using a simplified camera model, leading to less accurate results.

To obtain a better accuracy, non-linear methods have been developed [35] These approaches minimize a geometrical error, obtaining a non-linear objective function:

(12) $$\varepsilon (P)={\sum_{i=1}^{n}\left(\frac{p_{1}^{T}w_{i}}{p_{3}^{T}w_{i}}-u_{i}\right)}^{2}+{\left(\frac{p_{2}^{T}w_{i}}{p_{3}^{T}w_{i}}-v_{i}\right)}^{2}$$

This minimizing function represents the distance between the image points mi and the projection of the points w_i_ in the image, the so-called re-projection error. The next step is to compute the extrinsic parameters of the system (stereo calibration), relating each camera frame to a unique world coordinate system. (see Figure 2). The purpose of the stereo calibration is to find the relationship between the world coordinate system and the left and right camera coordinate systems:

(13) $${\text{X}}_{\text{cl}}=M_{l}{\text{X}}_{\text{w}}$$

(14) $${\text{X}}_{\text{cr}}=M_{r}{\text{X}}_{\text{w}}$$

where **M_l_** = [R_cl_, t_cl_] is the transformation matrix between the left camera coordinate system and the world coordinate system, **M_r_** = [R_cr_, t_cr_] is the transformation matrix between the right camera coordinate system and the world coordinate system, and **X_cl_**, **X_cr_**, **X_w_** represent the coordinates of the object point w in left camera, right camera and world coordinate systems respectively. The transformation matrices **M_l_** and **M_r_** represent rotations (R_cl_, R_cr_) and translations (t_cl_, t_cr_) between the world coordinate system and the left and right camera coordinate systems. The extrinsic parameters can be finally obtained through the same iterative process used to estimate the intrinsic parameters of each camera. It is based on the matching of correspondent points of the calibration sample in the image pair and consists of an overall optimization problem solving, that minimizes the re-projection error between camera planes.

## 4. Experimentation

Underwater imaging is seriously compromised by the turbidity of the medium, which decreases image contrast and attenuates light intensity, resulting in loss of details and colour alteration. Therefore, in this preliminary analysis, we have decided to conduct experimental tests in the laboratory using a water tank in order to control the lighting conditions and to ensure the repeatability and the reliability of measurements. The laboratory tests aim to analyse the effects of optical distortions, refraction and turbidity in close-range 3D reconstructions of objects for both the proposed techniques. In order to worsen the transparency of water (turbidity) and create a scattering media, different quantities of clay (with particles of about few micrometres in size) have been suspended in the water tank (2 × 3 m). In this way, along with the increase of the turbidity level, the loss of visibility increases and the ratio of light reflected by the objects decreases progressively. A calibration of a volume of about 400 × 300 × 200 mm has been performed at a distance of 1 m and two ceramic objects have been acquired. The object distance has been fixed by assessing the best compromise between overlapping of FOVs (cameras and projector) and brightness of the projector. The projector has been used as the only light source, so we have measured the illuminance with a luxmeter at the working distance in front of the system, as reported in Table 1, in order to provide a measurement of the lighting conditions at each different turbidity level. We have fixed the maximum value of the clay concentration at 20 mg/L, which required an exposure time of less than 10 s.

### 4.1. Underwater Setup

**Figure 4.**
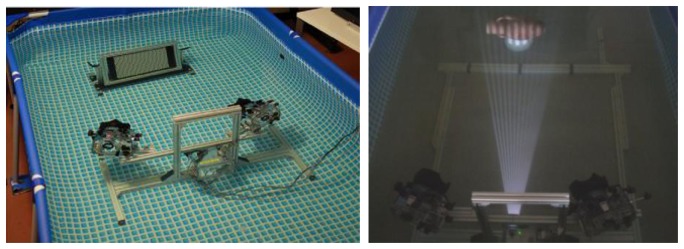
Optical setup of the underwater 3D acquisition system: underwater calibration (**Left**) and acquisition (**Right**).

Two Nikon D200 cameras, with a Charge Coupled Device (CCD) sensor size of 23.6 × 15.8 mm and a resolution of 3,872 × 2,592 pixels, equipped with an AF-Nikkor 35 mm lens, have been used to acquire the scene. The projector used in our experimentation is a Mitsubishi PK20, characterized by a very small size (123 × 48 × 97 mm), an acceptable resolution of 800 × 600 pixels with a brightness of 25 lumens and a low power consumption that allows for the use of a battery pack as power source. The three optical devices are protected by acrylic waterproof housings and fixed on an aluminium support; the optical setup is depicted in Figure 4. A dome port is mounted on the camera housing to reduce the refractive effects at the air-water interface [36].

**Figure 5.**
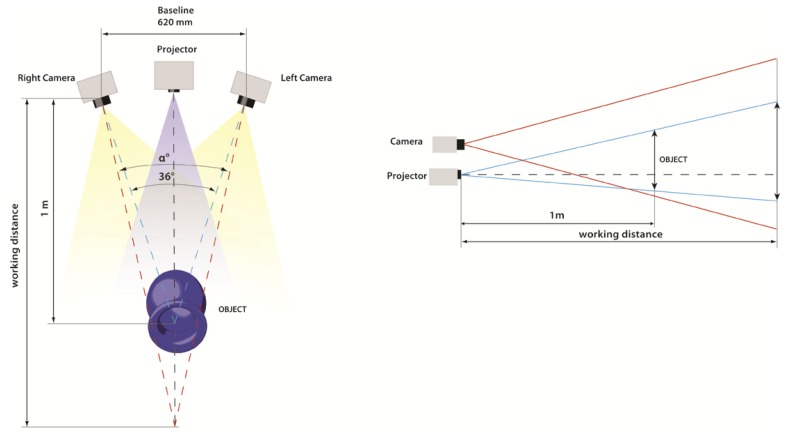
The optical configuration of the underwater 3D system can be adjusted by rotating and translating the cameras, in order to ensure the overlapping of the FOVs for the required working distance.

The system dimensions are 950 × 530 × 560 mm, with a weight of about 17 kg in air. The two cameras are mounted on the same bar, in order to have a 620 mm baseline (the distance between the optical centres of the cameras) and a 36 degrees angle between the optical axes. The projector is fixed on an independent bar in a lower position, in order to take into account the differences between the Fields-Of-View (FOVs) of the cameras and the projector (Figure 5). The latter generates an oblique off-axis projection, while the cameras present a symmetric FOV with respect to the optical axis. We chose this setup in order to allow for the acquisition of an object of 30–40 cm at a working distance of 1 m, maintaining a reduced scattering volume due to the overlap of the FOVs of the imaging devices.

The devices should be adjusted (rotation and vertical translation) in order to set up the acquisition at the chosen working distance, allowing the overlapping of the FOVs of cameras and projector as shown in Figure 5. The two cameras are remotely controlled by a PC through a USB interface connection, and the projection of the image sequence is controlled with an appropriate cable in order to optimize the acquisition time, manage the camera setting parameters and analyse the captured images in real time. The optical configuration of the system does not change for the active and passive techniques, therefore we can compare more effectively the results of 3D acquisitions obtained with the same camera positions, optical devices, calibration parameters and working distance. In particular, the same calibration procedure is performed in water for both techniques, computing the intrinsic and extrinsic parameters without changing the configuration of the cameras. The use of the same setup can ensure a better reconstruction accuracy without generating systematic errors due to multiple calibration procedures. In this way we can conduct a fair comparison between the two techniques.

**Figure 6.**
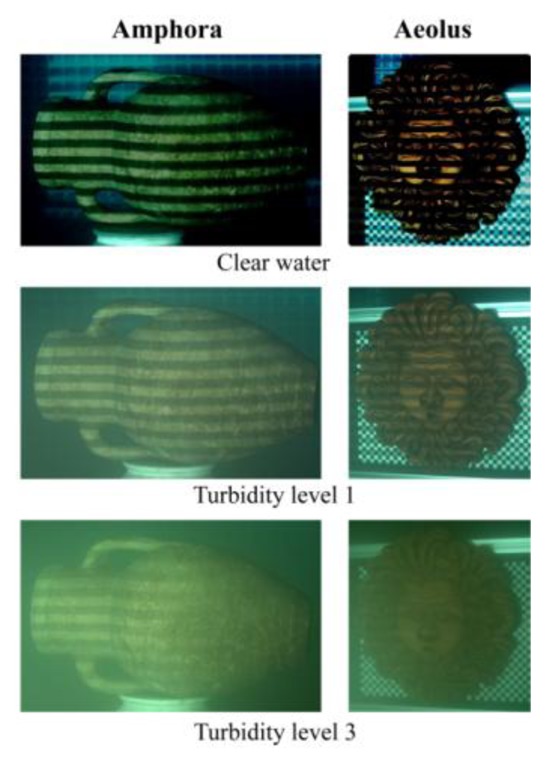
Images of the two objects with a projected grey-code pattern, acquired at different turbidity levels.

An amphora and a mask (Aeolus) have been acquired in air and water in this experimentation (Figure 6). For the passive stereo technique, only one image pair has been acquired by projecting a white pattern, while the active stereo technique needs at least 24 images to work. The images have been acquired in RAW format to ensure high quality and to extract each colour channel directly from RAW camera data. A 35 mm lens has been used to enclose the whole object in the camera FOV at a working distance of about 1 m. During the acquisitions, the aperture of the cameras was fixed to f/11 for a depth of field of about 100 mm, and the exposure time ranged from 0.8 s for the most luminous patterns, to 10 s for the darkest ones. The passive technique requires instead just 0.8 s for the projection of a white pattern to illuminate the scene. These long acquisition times are due to the use of the projector as the only light source, and to its low brightness.

### 4.2. Calibration

The calibration is performed in air and in water tank, in order to compare the results of intrinsic and extrinsic parameters obtained in both conditions and to evaluate its impact on 3D reconstructions. The calibration is conducted with a planar black and white checkerboard pattern (Figure 4) composed by 32 × 42 squares with an 8 mm side. This calibration sample has been placed in several poses and acquired by the two cameras in RAW RGB format in order to extract each channel (Red, Green, Blue) and perform a single colour calibration.

**Figure 7.**
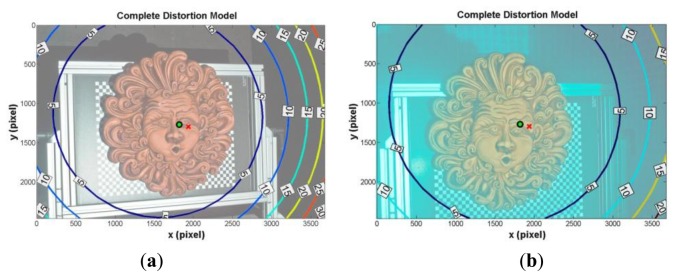
Complete distortion model (radial plus tangential distortions) in air and clear water. The cross and the circle indicate the image centre and the principal point, respectively. The contours represent the optical distortions values (in pixel), (**a**) Air; (**b**) Water.

The system has been first calibrated using the RAW camera files, extracting de-mosaicked grey scale images. The comparison between calibration parameters in air and water (Table 2) shows that the acrylic interface affects only the principal point coordinates that differ by 1 mm and 0.5 mm in the x and y directions, respectively, while focal length variation is equal to just 0.37 mm. This is due to a non-perfect alignment between the optical axes of camera lens and dome port, and to asymmetric components like the dome port shape. The comparison between the values in air and in water shows a difference on the focal length due to the refractive effect of 4.87 mm, which corresponds to an increase of about 13%. This variation of focal length implies a decrease of the FOV proportional to the medium index, because the CCD size is constant. Finally, the analysis of the principal point coordinates shows negligible variations. The complete distortion model, defined as the sum of radial and tangential components for each camera as depicted in Figure 7, shows the displacement of the principal point with respect to the image centre in x and y directions. The maps of optical distortions show that deviations are very small in the image centre where the object to be captured is located (maximum deviation at object border of about three pixels).

**Figure 8.**
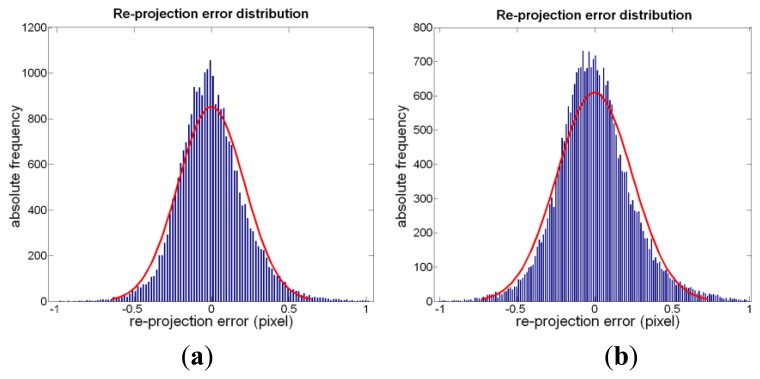
Distribution of the re-projection error (in pixels) in air and clear water (RGB images); (**a**) Air; (**b**) Water.

To evaluate the reliability of the underwater calibration, we considered the re-projection error for each corner point of the calibration checkerboard, defined as the distance between the points of the actual image, and those computed from the back projection of the ideal checkerboard. The error dispersions are Gaussian-distributed and the values of mean and standard deviation obtained in water, computed on both x and y directions, are comparable with the ones obtained in air (see Figure 8). This comparison allows us to consider that the assumed linear model of calibration in air can be used also in water, without taking into account the refraction effects.

Finally, in order to assess the effect of chromatic aberration, we have estimated the calibration parameters in the Red, Green and Blue colour channels. This optical phenomenon can cause blur in the images due to a degraded focus quality (longitudinal chromatic aberration) and a misregistration of the colour channels (transversal chromatic aberration), two factors that are difficult to reduce [37]. Despite this issue, the results have not shown significant differences in the values obtained by performing the camera calibration in water for each colour channel.

### 4.3. 3D Reconstruction—Passive Stereo

The dense stereo matching algorithm PVMS is used for the 3D reconstruction with the passive stereo technique. The algorithm inputs are an undistorted image pair and the 3 × 4 camera projection matrix, and the output is a coloured 3D point cloud. The camera projection matrix is computed from the same calibration parameters (intrinsic parameter matrix, rotation matrix and translation vector between left and right camera) of the stereo rig used for 3D reconstruction with the active technique. In order to correct the distortion of the image pair, we used a tool provided with the Camera Calibration Toolbox [32]. The PMVS parameters used for the fine tuning of the 3D reconstructions are the size of the correlation window and the level in the internal image pyramid used for the computation.

In our experimentation, we used a fixed correlation window with a size of 7 × 7 pixels, while the image resolution (image pyramid level) has been chosen accordingly to the turbidity level. A series of preliminary tests have been conducted in order to properly set up the algorithm parameters. In particular, for each turbidity condition, the value of the pyramid level k has been progressively reduced (the image is resized by a factor 2 k) from 1 to 3, maintaining the correlation window fixed to 7 × 7 pixels. Furthermore, the image pyramid level is fixed to 1, increasing the correlation window size from 7 × 7 pixels to 21 × 21 pixels. The results showed that as the turbidity increases, a wider correlation window is needed to overcome the loss of contrast due to the scattering effect. Otherwise, the same results can be obtained by using a fixed correlation window on a smaller image (with a higher pyramid level). A larger correlation window means higher computation time, hence, in order to preserve the reconstruction quality and save time, we used a small window of 7 × 7 pixels with k = 3 for the highest turbidity level, k = 2 for the medium turbidity level and k = 1 for low turbidity level.

### 4.4. 3D reconstruction—Active Stereo

The different image sets of the two objects have been processed to obtain the point clouds. In the active case, the software for 3D reconstruction is provided by Scansystems [38], so the 3D point cloud of the scene is computed from the 50 image pairs and the calibration data.

The 24 projected patterns allow us to exploit the maximum resolution of the projector (800 × 600) in order to obtain the maximum number of codified patches and, subsequently, the maximum number of 3D points. The lowest width of the bands of the projected pattern is four pixels, so the window of the codified patch is made up by about 20 pixels, if we refer to the used working distance. The reduction of the window size requires more patterns and a projector with a greater resolution, but this latter suffers also from a greater sensibility to the scattering.

On the basis of our preliminary investigations, where we reduced the width of the projected bands, we can assume that a minimum band of four pixels is a good compromise between the reduction of the scattering effect and the acquisition of a sufficient number of 3D points. In fact, we have verified that, if the reduction of the width of bands is too strong, the threshold algorithm applied to detect and separate the black and white bands on the object image in presence of turbidity (medium and high turbidity conditions) is not able to work properly.

## 5. Result Analysis

The point clouds computed through the active and passive techniques must be further elaborated to obtain the final 3D models, because many outlier points are present. Rapidform^®^ software has been used to edit the point clouds, while we used the open source software Cloud Compare [39] to perform measurements and comparisons on the 3D data. In this analysis, we aim to quantify the performance of both techniques by computing the density of 3D points, the percentage of outliers and the accuracy for the two objects. Moreover, a multi-channel analysis has been performed to evaluate the quality of 3D reconstructions by elaborating the images separately in each of the three colour channels.

### 5.1. Point Cloud Editing

**Figure 9.**
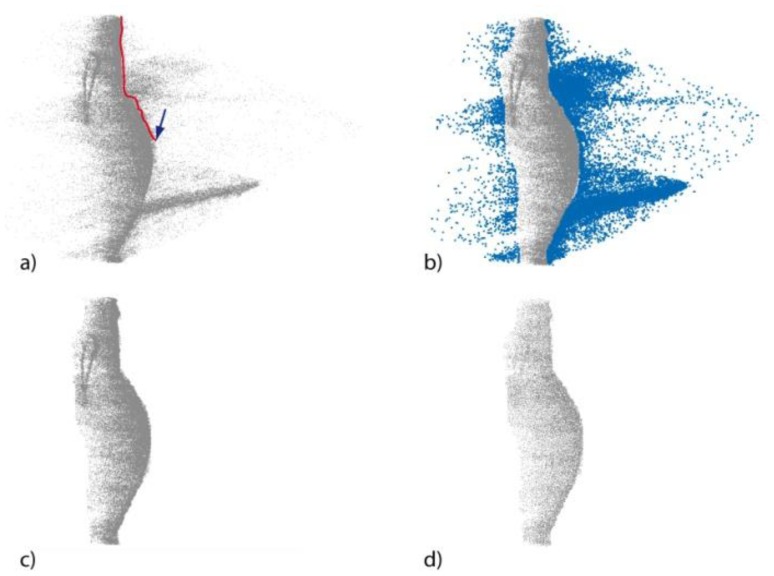
Point cloud cleaning procedure. Outlier point manual selection (**a**), selected outlier points (**b**), cleaned point cloud after outlier deletion (**c**) and final point cloud after noise filtering application (**d**).

**Figure 10.**
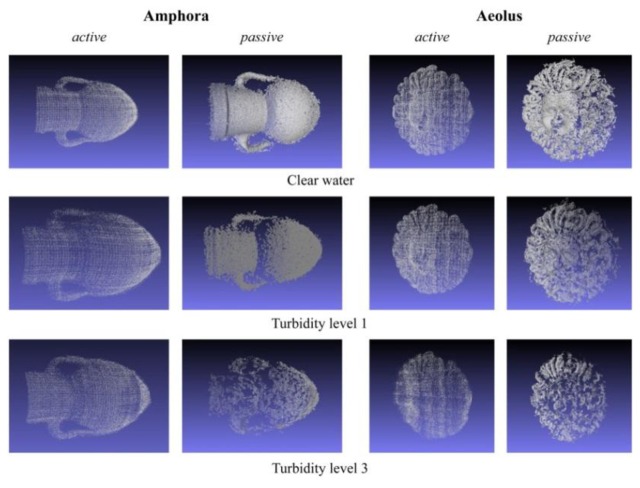
Point clouds obtained after editing procedure in different environment conditions for both active and passive techniques.

The first step consists in the manual deletion of outlier points that do not belong to the object surface (Figure 9a–c). This is the most time-consuming process, as it is highly dependent upon the surface complexity of the object to be processed. Whereas for a simple object, such as the amphora, only few steps with the lasso selection tool are required, a more complex surface requires manual and accurate intervention. In particular, once that a view of the 3D point cloud is chosen, a cursor is moved with the mouse on the screen to sketch a lasso (an arbitrary closed line) that allows for selecting a group of 3D points. The second cleaning step is the application of the noise filtering functions implemented in the software (Figure 9d), that delete automatically the noisy points close to the object surface. Figure 10 shows the points clouds acquired in different turbidity conditions with both the techniques after the editing procedure.

### 5.2. 3D Point Density

The density of 3D points acquired is computed by considering the ratio between the number of 3D points obtained after the editing procedure and the number of pixels that constitute the object in the image. The number of 3D points per 100 pixels (Np, Na for passive and active, respectively) are reported in Table 3 for both techniques. By comparing Np and Na in air, we found that the active technique returns a point density about three times lower than Np, as it depends on the projector resolution (800 × 600 in our case), while the passive technique depends on the image resolution. However, in medium turbidity (T2) conditions, this difference is reduced, because the passive technique is more affected by the turbidity which causes blur in the images. In fact, as we can see in Table 3, in the passive case the increase of turbidity causes a drastic decrease of the acquired points, while in active stereo the reduction of points is less noticeable, because this latter is less dependent on textures.

Moreover, it is important to analyse the percentage of points deleted during the preliminary editing procedure. The percentage values of deleted points (Npc% and Nac%) reported in Table 3 show that the active stereo technique is more affected by scattering, even under low turbidity conditions. Therefore, a time-consuming process is needed to manually delete the 3D points before using the noise filter, which works only on points that are very close to the object surface. The passive technique requires a lower noise reduction, but the scattering effect causes a loss of image contrast that compromises the matching of correspondent points and may generate holes in the reconstructed point clouds.

### 5.3. Multi-Channel Analysis

**Figure 11.**
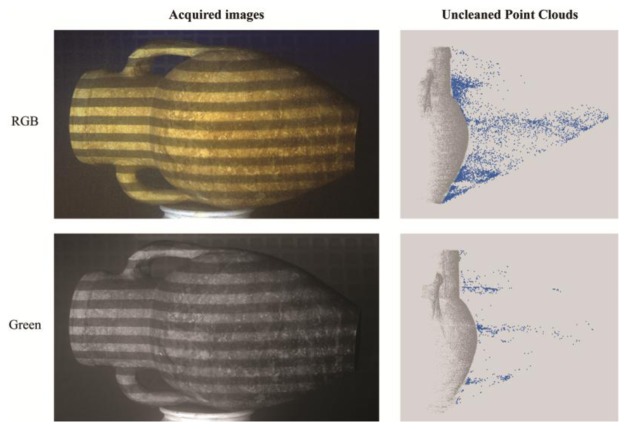
Acquired images and unclean point clouds at a light turbidity level (T1) in each colour channel: the blue points are due to scattering effects, which are highly reduced in the green channel.

It is common knowledge that in the underwater environment the absorption of light components varies with the depth, in particular the red component is completely absorbed at about 10 m, so the colours in the images of underwater scenes are altered and present some variations. However, in close-range capture this problem can be solved by using artificial light sources as strobes or lamps, and a multi-channel analysis can be performed in order to evaluate the quality of 3D reconstructions in the three separate colour channels. In fact, the contrast of the acquired images can be very different in the three channels, depending on the illumination and the object texture, so we had to check if the channel separation of light results in a spectral discrimination of component noise. The acquired RGB images used in the 3D reconstruction have been separated into the Red, Green and Blue channels and then processed. In the passive case, no significant differences have been observed, while in the active case substantial differences in the point density have been registered. As we can see in Figure 11, the outlier points are highly reduced when using the Green channel. The percentage of deleted points at turbidity level T1 has been reduced by about 66%, hence reducing the need for manual cleaning operation. Table 4 summarizes the results for the amphora object at each turbidity level, showing that the improvement ranges from about 40% (turbidity level T4) to about 70% (turbidity level T2). The results for the Aeolus mask present similar improvements, so they have been omitted.

### 5.4. Accuracy Evaluation

To evaluate the accuracy of the 3D reconstruction in water for both techniques, a series of experiments based on a statistical approach have been planned. First, two rectified samples, a plane (297 × 420 mm) and a cylinder (height 400 mm, radius 150 mm), have been acquired in clear water. These two samples allow us to quantify and compare the displacements of the acquired 3D points with respect to two known surfaces taken as references. The accuracy has been evaluated by comparing the 3D point clouds acquired in clear water with a cloud obtained in air and digitized through the active technique, which ensures high-accuracy reconstruction in air [38]. To measure the accuracy we have computed the mean distance μ (mm) among points over the entire cloud (distance between a point on one cloud and the closest point on the other cloud), using Cloud Compare software.

Table 5 contains the mean distance for the two samples and the related standard deviation σ (mm) obtained with the two techniques. The results lead us to consider that the two techniques show a very similar accuracy in water: we can note that the error is greater for a curved surface like the cylinder, given a mean distance of 0.29 mm and 0.20 mm for active and passive techniques, respectively. We can consider the values obtained for the flat surface (0.09 mm and 0.11 mm) as the maximum accuracy obtainable in water with the two techniques, respectively.

**Figure 12.**
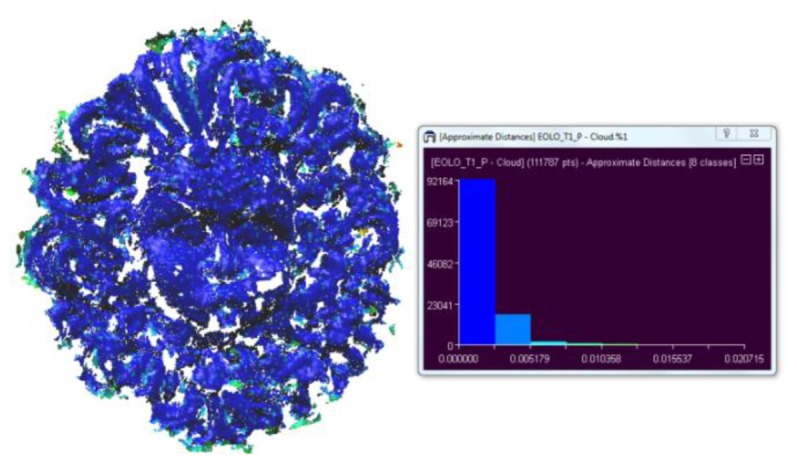
Map of mean distance distribution for the Aeolus mask.

In the second test, we have evaluated the accuracy at different turbidity levels for the Aeolus mask and amphora and compared the results with the ones obtained in air. A map of the mean distance distribution is shown in Figure 12, while the values of μ and σ are reported in Table 6 and Table 7. There is a slight increase in the standard deviation in presence of low and medium turbidity. When the environmental conditions are the same, the discriminant value is represented by the material properties: Aeolus shows a higher standard deviation caused by its dark texture. When comparing the μ data, we can assume that the accuracy depends on the texture of the objects and the turbidity conditions: we can conclude that the two techniques give comparable results, obtaining a geometrical error in high turbidity conditions (T3) lesser than 2.5 mm, a value that can be acceptable for many real world applications.

By comparing the results in clear water of Table 5, Table 6 and Table 7, it is apparent that the accuracy of the 3D reconstruction for both the techniques depends on the object shape; in particular, it increases considering a planar surface, a regular curved surface (Amphora) and an irregular surface (Aeolus).

In [40] the authors have analysed the refraction effects on the 3D reconstruction by multi-view acquisition, obtaining a minimum accuracy of 0.39 mm in clear water. This value can be compared with our results: with regards to the active technique, the value is the same, while for the passive case it is greater, even if included in the error range of the standard deviation.

### 5.5. 3D Point Cloud Integration

**Figure 13.**
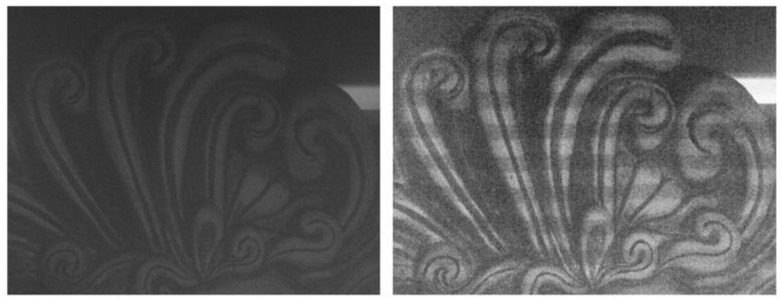
Dark and homogeneous areas present in the Aeolus image (**Left**) are better detected using structured light (**Right**), in the presence of turbidity.

By analysing the acquired point clouds (see Figure 10), we can confirm that the active technique is able to reconstruct the entire surface of the object in each condition and the point density is more or less constant over the surface as the 3D points are regularly distributed, while passive 3D reconstructions return a variable point density on the surface and are not able to reconstruct some portions. It is apparent that the active technique computes in each case the 3D point coordinates of the codified scene, while the passive one computes only the 3D coordinates of matched points in the image areas where features are found. In particular, as shown in Figure 13, it is difficult to find features in dark and homogeneous areas with the passive technique, while the codification of image points may be improved by projecting structured-light patterns. Obviously, in absence of other information, void parts must be reconstructed in post-processing, with the inevitable possibility of errors. For this reason we propose an integration of 3D point clouds acquired with the two approaches, exploiting the advantages of each technique: the better point density of the passive technique and the better coverage of the object surface of the active technique.

**Figure14.**
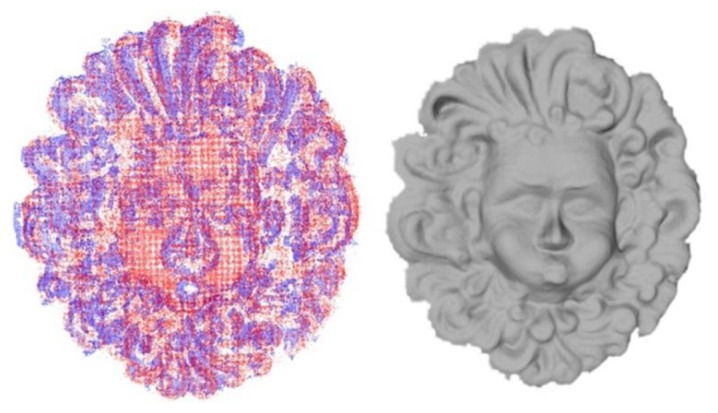
(**Left**), comparison between the two point clouds of the Aeolus mask obtained with passive (blue points) and active technique (red points), respectively; (**Right**), triangular mesh of the Aeolus mask obtained from the integrated point cloud.

A comparison of two point clouds acquired with the two techniques is shown in Figure 14, where it is possible to recognize the areas that are not reconstructed by the passive technique. The union of the two point clouds is performed with Rapidform software (Combine tool) in order to obtain a single point cloud. Then we apply a redundancy filter in order to delete the closest points by setting an automatic threshold value, and finally a triangular mesh can be created (Figure 14). This resulting point cloud has been compared with the one acquired in air (as in Table 7) for the T3 case and we have obtained a mean distance of 1.95 mm with a standard deviation of 1.75 mm. The point density is increased at 4.7 points per 100 pixels. With respect to each individual technique, the error has decreased as a greater number of closer points have been found.

## 6. Discussion

The experimental setup proposed in this work is not suitable for tests in real marine conditions and applications (handling by a scuba diver or installation on a ROV). In fact, a series of additional considerations must be taken into consideration with regards to the presence of moving elements (flora and fauna), the colour alteration, the corrosion of the object surface, the instability of the imaging system and the synchronization of the camera shutters. The purpose of our system is to perform a fair comparison between two 3D techniques based on image acquisition that use two different approaches to reconstruct 3D models. Moreover, some hardware modifications must be taken into account for the lighting sources (wavelengths and illuminance) in order to improve the image acquisition phase and reduce the required time. We neglect the variation of absorption of colour spectrum in marine environments, because we have conducted the acquisitions in the laboratory using just an artificial light source.

Therefore, a deep analysis of the real condition effects must be done and a new design of experimentations in real cases is required—we hope to present them in a future work. We can consider the results of this experimentation as reference data which we will compare to the ones to be conducted in a true marine environment. For example, we could understand if the calibration parameters can be computed in laboratory with a water tank avoiding a calibration procedure *in situ*, or if it is necessary to include refraction and scattering effects in the optical model of image formation. However, we have found that the two techniques gave comparable results and the 3D data integration can be a solution to improve the 3D acquisition quality in terms of coverage of the acquired scene and stability in turbidity conditions. In particular, the projection of patterns on the scene can help the reconstruction of areas with scarce contrast and natural features. For this purpose, a series of possible solutions to facilitate the application in real cases can be proposed as follows:

- using the fringe projection technique that requires the projection of just one pattern, considerably reducing the acquisition time;
- projecting a coloured pattern to increase the image contrast of the acquired pattern;
- projecting a fringe pattern using a grating fixed on the flash lamp handled by a scuba diver;
- employing a more powerful light source;
- studying, with a multi-channel approach, the separation of the projected pattern from the texture acquired simultaneously with one shot, in order to apply active and passive techniques and perform the 3D data integration.

## 7. Conclusions

We have proposed a preliminary comparison between two 3D imaging techniques based on active and passive approaches. The experimentation has been conducted in the laboratory in a water tank, controlling the lighting conditions in order to test the performances of both techniques in terms of accuracy and density of the acquired 3D points. 3D acquisitions have performed under poor visibility conditions, obtained by suspending different concentrations of clay in water, which allow us to take into account the scattering effects, *i.e.*, the main limitation in underwater imaging. For a fair comparison, we have adopted a 3D system in stereo configuration, composed by two cameras and a projector lodged in waterproof housings. Therefore, the acquisition process is performed for both the techniques with the same experimental setup, working distance, lighting conditions and calibration parameters. This setup allows us to align automatically the acquired point clouds, because these are computed with respect to the same coordinate system.

The results of calibration show that a linear model can be employed in 3D underwater acquisition, without taking into account the refractive index of media, with an acceptable accuracy. The point clouds obtained with the active technique give more stable results in presence of turbidity, due to the use of coded patterns to compute the 3D point coordinates, but they are affected by a noticeable noise level due to scattering even in low turbidity, thus requiring some manual operations to clean the point cloud. To reduce this problem we have investigated the use of a single colour channel for the 3D reconstruction, finding that the acquisition of underwater images in the Green channel may give good improvements in terms of noise reduction and surface accuracy. The passive technique gives a cleaner and denser point cloud in clear water with low turbidity levels, but the results are strongly related to the surface texture of the acquired object. In fact, in low-textured areas, the loss of contrast due to turbidity results in holes and missing areas. Moreover, we propose an integration of 3D point clouds acquired with the two approaches, exploiting the advantages of each technique: the better point density of the passive technique and the better coverage of the object surface of the active technique. The 3D point cloud resulting by the integration allows for the improvement of accuracy and density.

Finally, the proposed 3D system has been designed for laboratory tests and at the moment is not suitable for real world applications. In future works, we will design a new experimentation layout to test the two techniques also in a marine environment, taking into account more factors such as the variable lighting conditions, the surface corrosion, the system instability, the synchronisation of the cameras and more. For the real tests, we will probably employ the fringe projection technique, because it allows for decreasing drastically the required time for the acquisition. Our results can be used as a reference for further comparisons in the analysis of other 3D techniques and algorithms.

## Figures and Tables

**Table 1.** Measurements of the turbidity conditions.

**Turbidity Level**	**Clay Used (mg/L)**	**Illuminance (lux)**
Clear Water	0	96.7
Turbidity level 1(T1)	5	80.4
Turbidity level 2 (T2)	10	70.5
Turbidity level 3 (T3)	15	44.6
Turbidity level 4 (T4)	20	21.5

**Table 2.** Intrinsic parameters (mean values for left and right camera) in air and water (RGB images).

**Parameter (mm)**	**Air**	**Water**
Focal length	37.19	42.43
Principal point px	12.23	11.04
Principal point py	8.22	7.42

**Table 3.** Acquired 3D points per 100 pixel (Np for passive and Na for active stereo) and percentage values of deleted 3D points (Npc% for passive Nac% for active stereo) for Amphora and Aeolus.

	**Amphora**				**Aeolus**			

**Condition**	**Np**	**Npc%**	**Na**	**Nac%**	**Np**	**Npc%**	**Na**	**Nac%**
Air	13.0	1.93	3.4	4.52	11.9	0.98	3.2	3.17
CW	14.3	2.79	3.9	17.50	13.8	0.94	3.5	22.61
T1	14.2	4.23	4.1	23.96	3.8	3.53	3.2	24.93
T2	3.8	5.07	3.3	34.58	2.8	9.93	2.9	20.50
T3	2.9	6.35	3.5	30.63	2.5	3.21	1.7	42.81
T4	0.2	20.42	2.2	47.99	-	-	-	-

**Table 4.** Percentage values of deleted 3D points for active stereo technique in each colour channel, Red (Nac%-R), Green (Nac%-G) and Blue (Nac%-B), compared to the values computed from RGB images (Nac%-RGB). The table is related to the object Amphora.

**Condition**	**Na RGB**	**Na R**	**Na G**	**Na B**	**Nac %-RGB**	**Nac %-R**	**Nac %-G**	**Nac %-B**
Air	3.4	3.3	3.3	2.8	4.52	3.49	2.67	17.02
CW	3.9	3.8	4.4	4.0	17.50	12.33	6.45	11.72
T1	4.1	4.4	5.2	3.9	23.96	11.33	8.09	16.09
T2	3.3	3.0	2.4	2.0	34.58	15.84	9.26	16.11
T3	3.5	3.6	2.3	1.5	30.63	23.55	16.56	36.75
T4	2.2	2.1	1.2	0.5	47.99	29.03	28.10	58.56

**Table 5.** Accuracy evaluation on a planar and cylindrical sample in clear water (mm).

	**Plane Sample**		**Cylinder Sample**	
		
	**Passive**	**Active**	**Passive**	**Active**
*μ*	0.11	0.09	0.20	0.29
*σ*	0.08	0.07	0.13	0.25

**Table 6.** Amphora: accuracy evaluation (mean distance μ in mm and standard deviation σ).

**Condition**	**Active**		**Passive**	
	
	**μ**	**σ**	**μ**	**σ**
CW	0.40	0.15	0.22	0.55
T1	0.44	0.23	0.52	0.65
T2	0.47	0.23	0.69	0.78
T3	0.75	0.66	0.85	0.87
T4	0.86	0.88	2.23	1.86

**Table 7.** Aeolus: accuracy evaluation (mean distance μ in mm and standard deviation σ).

**Condition**	**Active**		**Passive**	
	
	**μ**	**σ**	**μ**	**σ**
CW	0.75	0.81	0.70	1.06
T1	1.61	1.26	1.53	0.90
T2	1.98	1.56	2.08	1.49
T3	2.32	1.98	2.44	2.00
T4	2.83	2.81	-	-
